# A Fatal Case of Placenta Præmia

**Published:** 1873-02

**Authors:** G. C. Paoli

**Affiliations:** Chicago


ARTICLE III.—*A Fatal Case of Placenta Prævia.* By G. C. PAOLI, M.D., Chicago.

On New Year's day, at 4 o'clock P. M., I was requested to call to see a case of midwifery, complicated with hemorrhage of the uterus.

On my arrival I found Mrs. C., twenty-five years of age, previously the mother of two children, under strong labor pains; she was nearly pulseless, with cold extremities, cadaverous countenance, and in a state of extreme exhaustion. She was tossing about in the bed in her efforts to breathe.

On vaginal examination, I found the uterus well dilated, but very hard to the touch; and besides, a fleshy fibrous mass, which indicated a placental presentation. Previous to my arrival, the liquor amnii had escaped. I gave my patient at once a stimulant, consisting of spiritus terebinthina, and ordered the room to be warmed up, as it was rather cool when I entered.

I placed the patient on the edge of the bed, so that I could easily introduce my hand in the vagina for the purpose of separating the placenta, which was firmly attached to the uterus. After some careful manipulations, I succeeded in breaking up the adhesion, and discovered a breech presentation in grasping one of the feet. I extracted a male child, apparently eight months old, who, no doubt, had been dead a couple of days. There was scarcely any loss of blood during the operation. I placed her at once in a horizontal position, and with blankets dipped in warm water around her body, but I could not by any means get up any reaction.

The woman died about half an hour after delivery, in a state of syncopation.

*Remarks on the Case.*—The woman had been bleeding, more or less, for two months. Dr. C., who was called to see her, before Christmas, stated to her husband that it was not dangerous, and that he had often observed such cases in the course of his praxis. But as both the woman and her husband became alarmed by the occasional gushing of blood, they sent for Dr. B., who, after examining her, stated that it would not be professional for him to undertake the case, as he would be liable therefor to be expelled from any medical society.

